# Medical Management of Thyroid Ectopia: Report of Three Cases

**DOI:** 10.4274/Jcrpe.1103

**Published:** 2013-09-18

**Authors:** Deep Dutta, Manoj Kumar, Anubhav Thukral, Dibakar Biswas, Rajesh Jain, Sujoy Ghosh, Satinath Mukhopadhyay, Subhankar Chowdhury

**Affiliations:** 1 IPGMER & SSKM Hospital, Department of Endocrinology & Metabolism, Bose Road, Calcutta, India

**Keywords:** Thyroid ectopia, lingual thyroid, submandibular thyroid, thyroglossal cyst thyroid, dual ectopic thyroid, levothyroxine

## Abstract

Thyroid ectopia (TE) is an embryological aberration of the thyroid gland migration most commonly observed in the lingual region followed by the sublingual, hyoid, and mediastinal regions. TE is often complicated by local compressive symptoms resulting in dysphagia, dysphonia, and dyspnea. Surgical removal of TE is frequently complicated by difficulties in intubation, increased perioperative bleeding, and severe primary hypothyroidism; on the other hand, I131 ablation is limited by high doses needed and the concern for long-term effects especially in children. We report three children with TE who all presented with compressive symptoms and were managed conservatively with levothyroxine resulting in resolution of compressive symptoms and favorable outcomes. Levothyroxine supplementation is effective and has an important role in managing TE, not only in correcting the associated hypothyroidism but also in resolving the associated compressive symptoms by reducing the size of the ectopic thyroid tissue.

**Conflict of interest:**None declared.

## INTRODUCTION

Thyroid ectopia (TE) is an embryological aberration of thyroid migration characterized by presence of thyroid tissue at sites other than its normal location, most commonly in the lingual region (90%; incidence 1:4000-1:10000), followed by the sublingual, hyoid/pre-laryngeal, and mediastinal regions (1,2). Other rare sites include the pharynx, oesophagus, trachea, lung, heart, breast, duodenum, mesentery, and adrenal gland (1,2,3). Observed most commonly in children and adolescents (male/female=4/1), TE is usually functionally incapable of meeting physiologic requirements resulting in hypothyroidism in 33-62% of these individuals (4). TE in the lingual, sublingual or laryngeal region may present with localized swelling and compressive symptoms resulting in dysphagia, dysphonia, and dyspnea often necessitating surgical removal which is frequently complicated by difficult intubation and increased peri-operative bleeding (5). We present three cases of TE with compressive symptoms all managed conservatively with favorable outcomes.

## CASE 1

An 8-year-old boy (height 122 cm; 25-50th percentile; weight 20.5 kg; 25-50th percentile) presented with snoring which had started at age 5 years and had increased over time. Complaints of dysphagia and odynophagia had started six months ago, at which time the patient was detected to have a mass (3X2 cm reddish swelling with smooth surface; Figure 1a) at the base of his tongue, demonstrating an increased uptake on 99mTc scan and being consistent with lingual thyroid. No uptake was noted in the thyroid bed (Figure 1b). Ultrasonography (USG) of the neck revealed absence of thyroid tissue in the normal position in the neck and confirmed the presence of lingual thyroid. Hormonal evaluation revealed a state of subclinical hypothyroidism (Table 1). Levothyroxine, administered in a dose of 75mcg/d for one year, resulted in reduction of the swelling along with resolution of the dysphagia and odynophagia (Figure 1c). Levothyroxine was continued in a dose of 50mcg/d.

## CASE 2

An 18-month-old girl patient (height 70 cm; 5-10th percentile; weight 9.5 kg; 10-25th percentile) presented with complaints of poor feeding and of a swelling in the submandibular region, which moved with deglutition, noted 6 months ago (Figure 2a). The swelling was detected to be a 4X3 cm mass at the base of the tongue which showed increased uptake on 99mTc scan, consistent with lingual thyroid, without any uptake in thyroid bed (Figure 2b). The patient was also shown to have primary hypothyroidism (Table 1). Following one year of levothyroxine therapy at 50mcg/d led to resolution of feeding problems along with a decrease in the size of the swelling.

## CASE 3

Primary hypothyroidism was diagnosed in a 5.5-year-old girl patient (height 105 cm; 25th percentile; weight 17 kg; 25-50th percentile) who presented with complaints of dysphagia, poor feeding, constipation, and dry skin in the past six months and a midline neck swelling moving with deglutition noted at age 3 years (Figure 3a). The patient had an increased 99mTc uptake suggestive of functional thyroid tissue in a thyroglossal cyst. A second area of uptake was noted in the submandibular region along with normal uptake in the thyroid bed (Figure 3b). These findings were confirmed by USG (Table 1). A diagnosis of dual ectopic thyroid (thyroglossal cyst and submandibular region) with eutopic thyroid was made. Symptoms subsided and dysphagia resolved with levothyroxine given in a dose of 50mcg/d and increased to 75mcg/d over nine months.

## DISCUSSION

Aberrant descent of thyroid anlage from the floor of the primitive hypopharynx is the cause for TE and the associated absence of eutopic thyroid in ~70% of the cases (6,7,8). In our series of three patients, eutopic thyroid was present in only one case. Lingual thyroid was documented in two patients, and oral examination played an important role in its diagnosis. Any patient with a midline swelling in the head and neck region, below the base of tongue should undergo thyroid scintigraphy to rule out TE. Thyroglossal cysts, which are remnants of the thyroglossal duct are the most common midline neck mass in childhood (>75%) with 35-70% containing thyroid tissue (9,10). Hence, thyroid scintigraphy is mandatory in the evaluation of a thyroglossal cyst, especially when solid tissue is palpable or documented on imaging. Malignancy is rare in thyroglossal cysts (found in <1% cases) and is almost always papillary carcinoma (11).

Computed tomography (CT) and magnetic resonance imaging (MRI) are helpful in better anatomic delineation of the lesion, however are not routinely required for diagnosis since the same information can be obtained by clinical examination, thyroid scintigraphy and USG. Use of CT/MRI may be limited to difficult cases where surgical removal is contemplated.

Presence of two ectopic foci of thyroid tissue in the same patient (dual ectopia) is extremely rare with only a few isolated reports (12,13). In most patients of dual ectopia, one of the foci is usually lingual/sublingual thyroid and the other is subhyoid/suprahyoid thyroid (12,13). Presence of dual ectopia along with a eutopic thyroid as observed in Case 3 is even rarer, with perhaps only a single report to date (8).

TE carries a low risk of malignancy (<1%) and this is a point in favor of conservative management (8,14). Levothyroxine treatment is usually warranted for several months before contemplating for alternatives. I131 ablation is increasingly being shown to have a beneficial role in managing compressive symptoms of TE in hyperthyroid, euthyroid, and hypothyroid individuals (15,16). Limitations of I131 therapy include the need for high doses to induce regression (~20 mCi or more), especially in euthyroid and hypothyroid individuals. I131 therapy is generally avoided in children and young adults due to its potential unknown long-term effects (15,16,17). Surgery should be reserved for patients not responding to levothyroxine treatment and those presenting with severe compressive/obstructive symptoms or bleeding.

To conclude, it may be said that levothyroxine supplementation has an important role in managing TE, not only in treating hypothyroidism but also in resolution of its compressive symptoms by reducing the size of the ectopic gland.

## Figures and Tables

**Table 1:**
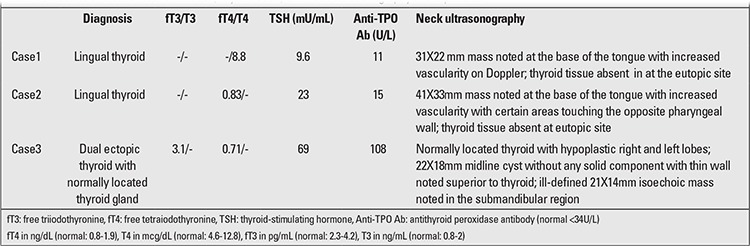
Results of biochemical evaluation, thyroid scan, and neck ultrasonography in the patients

**Figure 1a:**
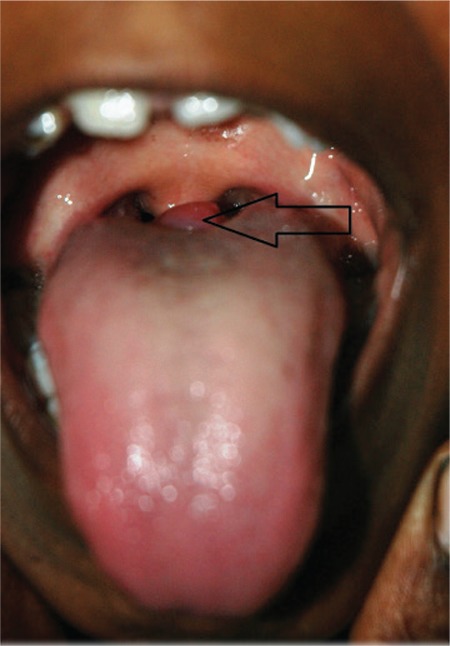
Oral examination of Case 1 showing swelling at the base of the tongue (black arrow) suggestive of lingual thyroid

**Figure 1b:**
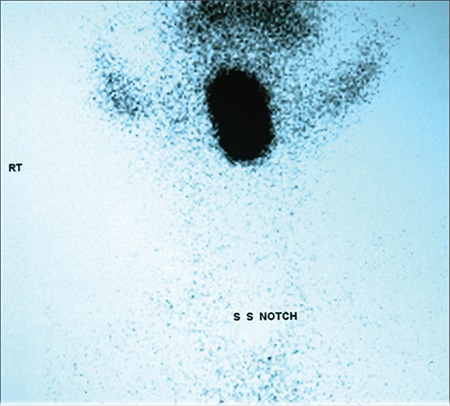
Sodium pertechnetate scan showed increased 99mTc uptake at the base of the tongue, consistent with lingual thyroid, without any uptake in the thyroid bed in Case 1

**Figure 1c:**
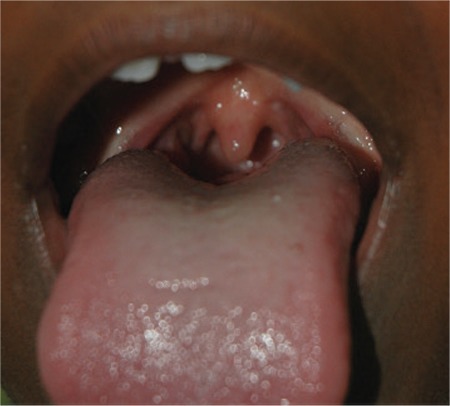
Significant reduction in the size of lingual thyroid following 1 year of levothyroxine therapy

**Figure 2a:**
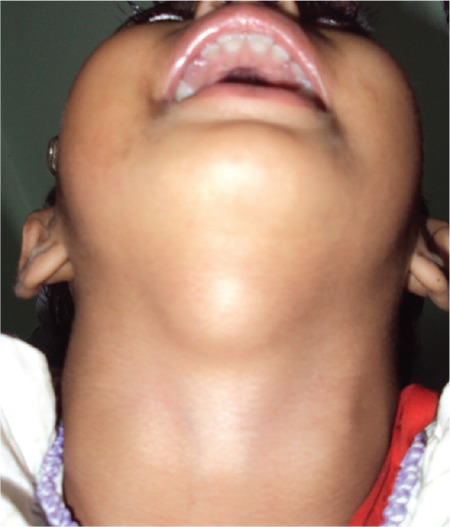
Swelling in the submandibular region externally along with visibility at the root of the tongue suggestive of lingual thyroid

**Figure 2b:**
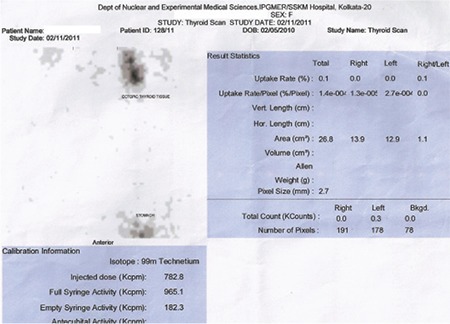
Sodium pertechnetate scan showed increased 99mTc uptake in the region of swelling suggestive of lingual thyroid, without any uptake in the thyroid bed in Case 2

**Figure 3a:**
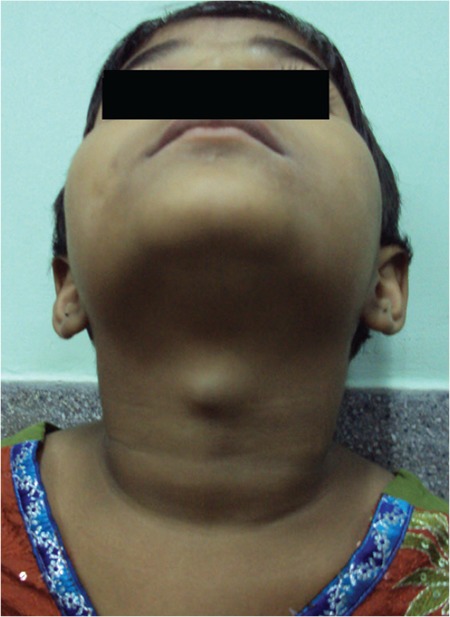
Midline cystic swelling superior to the thyroid cartilage with tongue protrusion suggestive of thyroglossal cyst in Case 3

**Figure 3b:**
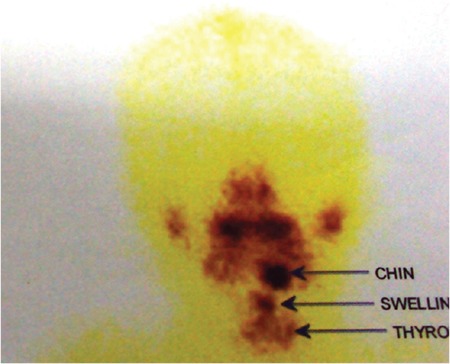
Sodium pertechnetate scan showed increased 99mTc uptake in the region of the thyroglossal cyst suggestive of functional thyroid tissue in the cyst, a second area of uptake in the submandibular/chin region along with normal uptake in the thyroid bed suggestive of presence of eutopic thyroid in Case 3
